# Success of Using Iodopovidone as a Sclerosing Agent for Chemical Pleurodesis in a Tertiary Care Center: A Descriptive Cross-Sectional Study

**DOI:** 10.31729/jnma.6033

**Published:** 2021-01-31

**Authors:** Deebya Raj Mishra, Narendra Bhatta, Puru Koirala, Bhupendra Shah, Bidesh Bista, Niharika Shah

**Affiliations:** 1 Department of Pulmonary, Critical Care and Sleep Medicine, B.P. Koirala Institute of Health Sciences, Dharan, Nepal; 2 Department of Internal Medicine, Birat Medical College, Nepal; 3 Department of Internal Medicine, B.P. Koirala Institute of Health Sciences, Dharan, Nepal; 4 Department of Internal Medicine, Civil Hospital, Nepal; 5 Department of Pathology, B.P. Koirala Institute of Health Sciences, Dharan, Nepal

**Keywords:** *iodopovidone*, *pleurodesis*, *pneumothorax*

## Abstract

**Introduction:**

Pleurodesis is a procedure to achieve symphysis between the two layers of pleura aimed at preventing the accumulation of either air or fluid in the pleural space. In Nepal, intrapleural instillation of the chemical sclerosing agent is more commonly done as a Thoracoscopy facility is not easily available. However, iodopovidone is rarely used for this purpose in Nepal. The study aims to find the prevalence of success using iodopovidone as the chemical sclerosing agent.

**Methods:**

The study included cases undergoing pleurodesis over a two-year period. The clinic-odemographic data, diagnosis, treatment effect and treatment response were analyzed. The treatment response was graded as Treatment Success (Complete Response or Partial Response) and Treatment Failure.

**Results:**

Pleurodesis was done in a total of 54 cases. Of those, 39 cases were Secondary Spontaneous Pneumothorax, 11 were Malignant Pleural Effusion, 3 were Primary Spontaneous Pneumothorax, and 1 was a case of Hepatic Hydrothorax. Among Secondary Spontaneous Pneumothorax, Pleurodesis was successful in 37 (95%) out of 39 cases, with 35 (90%) having a Complete Response and 2 (5%) having a Partial Response while 2 (5%) had Treatment failure. Among Malignant Pleural Effusion, treatment success was achieved in 6 (55%) out of 11, whereas 5 (45%) failed the treatment. The commonest complication was burning sensation, and the commonest pain scale was “distressing.”

**Conclusions:**

This study highlights the safety and the ease of use of iodopovidone as an agent for chemical pleurodesis. It confirms the high rate of success of pleurodesis in cases of pneumothorax as found in other studies. In contrast, the success rate is understandably lower in cases of Malignant Pleural effusion.

## INTRODUCTION

Pleurodesis is a procedure to achieve symphysis between the two layers of pleura aimed at preventing the accumulation of either air or fluid in the pleural space.^1^ It is performed in procedures such as Malignant pleural effusion and pneumothorax. Pleurodesis can be performed in three ways, i.e., intrapleural instillation (through a conventional chest tube thoracostomy) of sclerosing chemical agent, surgical intervention via a standard thoracotomy and more recently, thoracoscopic surgery and pleurodesis.^2^

Thoracoscopic surgery though less invasive, is not available freely in every hospital and is also not cost-effective.^3–5^ Moreover, conventional pleurodesis is easy, cheap and safer than surgery.^3,4^

In Nepal, consistent with our institution's practice, intrapleural instillation of chemicals is done as a thoracoscopy facility is not easily available. Moreover, most centers are still using Tetracycline derivatives, esp. Oxytetracycline. The evidence for the use of Oxytetracycline is scarce. Initially, parenteral Tetracycline was being used, but clinicians started using parenteral Doxycycline and Minocycline after its production stopped in the 1980s. However, since both these agents are not available in our country, the use of Oxytetracycline has continued.

Iodopovidone is a cheap and easily available agent with comparable success as a chemical sclerosing agent for pleurodesis.^6^ Thus, the study was initiated to explore the success of chemical pleurodesis with the use of iodopovidone in our setting.

## METHODS

Patients were enrolled over two years and were followed up for at least a period of one year. Ethical approval was obtained from the Institutional Review Committee (IRC/0892/016). The following consenting cases were included consecutively for pleurodesis;

1. Recurrent primary spontaneous pneumothorax
2. First episode of secondary spontaneous pneumothorax
3. Malignant pleural effusions large enough to cause dyspnea and not to respond to treatment of the underlying tumor and to require palliative therapy directed at the pleural space
4. Undiagnosed cases of recurrent pleural effusions or other causes as last resort of symptomatic treatment.

The procedure for chemical pleurodesis with iodopovidone was as follows;

First, the patient was given an intravenous analgesic like Tramadol. Following that, the pleura was anesthetized with 2mg/kg of 2% Xylocaine solution. Then, a mixture of 20 mL of 10% topical solution of povidone-iodine and 80 mL of normal saline was instilled into the pleural cavity through a thoracostomy tube. Then the tube was clamped for 2 hours. The position of these patients was changed within 2 hours to circulate the mixture. After unclamping, the thoracostomy tube was removed as soon as the drainage decreased to <100 mL per day.

The extubation was followed by chest-x-rays with repeat x-rays at two weeks, and follow-up data until 12 months were reviewed.

The response to this procedure was graded as treatment success and treatment failure. Treatment success included both a Complete Response or a Partial Response. These were defined as;

“Complete Response” as a complete radiographic resolution of

a. Pneumothorax: detected as the disappearance of the visceral line on Erect PA Chest X-ray.
b. Pleural effusion: detected as the disappearance of initially present homogeneous opacity on Erect PA Chest X-ray.

“Partial Response” as a recurrent pleural disease that did not require additional thoracentesis/ repeat chest tube placement.

“Treatment Failure” as a recurrent pleural disease that required repeat thoracentesis/ repeat chest tube placement.

The distinction between partial response and treatment failure was based on the improvement of clinical signs and symptoms in the patient and the rapidity of reaccumulation and the need for repeat thoracentesis or repeat chest tube placement.

The patient's pain response was analyzed by assessing the recorded “Visual Analog Scale” and Wong-Baker FACES scale. The pain response was evaluated before pleurodesis, 15 minutes and 2 hours after pleurodesis once the tube was unclamped.

Descriptive statistical analysis was done from the demographic data of patients. Results were expressed as mean ± SD. All statistical analyses were performed with computer-based analysis software, SPSS version 17.0.

## RESULTS

Fifty-four patients underwent Chemical Pleurodesis with Iodopovidone during the two-year study period. Treatment success was achieved in 37 (95%) cases with 39 secondary spontaneous pneumothorax and 6 (55%) cases with 11 malignant pleural effusion.

Of the 54 patients included, 44 (82%) of the patients were male. Four (7%) of the patients were less than 35 years old, whereas 21 (39%) were between the ages of 35 to 60, and the majority, 29 (54%), were more than 60 years of age.

The commonest cause for pleurodesis was Secondary Spontaneous Pneumothorax 39 (72.2%). There were 11 patients in whom pleurodesis was done in 54 patients secondary to Malignant Pleural Effusion, and there was one case of Hepatic Hydrothorax (Table 1).

**Table 1:** Clinical Diagnosis

Primary Diagnosis	n (%)
Primary Spontaneous Pneumothorax (n=3)	3 (5.55)
Secondary Spontaneous Pneumothorax (n=39)	
COPD	21 (38.88)
Post TB sequelae	18 (33.33)
Malignant Pleural Effusion (n=11)	
Bronchogenic Carcinoma	7 (12.96)
Breast Carcinoma	2 (3.70)
Gastric Carcinoma	1 (1.85)
Unknown	1 (1.85)
Hepatic Hydrothorax (n=1)	1 (1.85)
Total	54 (100)

Among the 39 cases of Secondary Spontaneous Pneumothorax, treatment success was achieved in 37 (95%) with Complete Response being achieved in 35 (90%). In contrast, Partial Response was achieved in 2 (5%), and the treatment failed in another 2 (5%) of the cases. In one of the cases in which treatment failed, pleurodesis was attempted thrice (Table 2).

**Table 2:** Treatment Response to Pleurodesis with Iodopovidone.

Diagnosis	Treatment Success		Treatment Failure
	Complete Response n (%)	Partial Response n (%)	
Primary Spontaneous Pneumothorax (n=3)	3 (100)	-	-
Secondary Spontaneous Pneumothorax (n=39)	35 (90)	2 (5)	2 (5)
Malignant Pleural Effusion (n=11)	6 (55)	-	5 (45)
Hepatic Hydrothorax (n=1)	1 (100)	-	-

Among the 11 cases of Malignant Pleural Effusion, Complete Response was achieved in 6 (55%), whereas the treatment failed in 5 (45%) (Table 2).

Complete response was achieved in all the cases of Primary Spontaneous Pneumothorax and Hepatic Hydrothorax (Table 2).

The commonest complication of Chemical Pleurodesis was a burning sensation, which was present in 42 (78%), followed by dyspnea in 10 (19%) and fever in 2 (4%). In both episodes, fever was transient and responded to a single dose of oral antipyretic.

The pain of different gradations was present in all the cases. The pain was measured with the Wong-Baker Faces pain scale at 15mins and 2 hours after pleurodesis, and the response was as given in (Table 3)

**Table 3:** Wong-Baker FACES scale–Pain Scale.

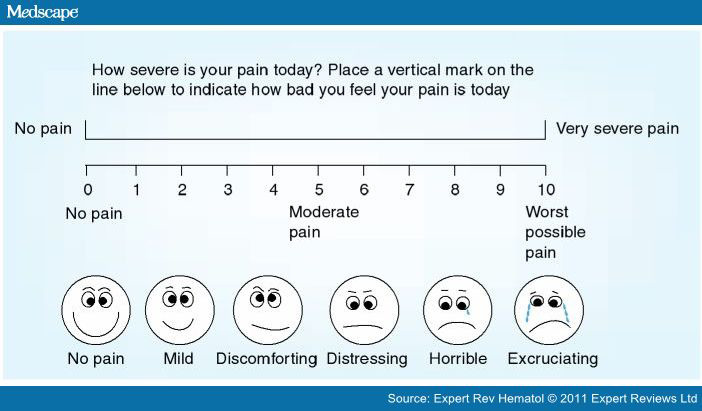						
	No Pain	Mild	Discomforting	Distressing	Horrible	Excruciating
At 15 mins	0	5 (10%)	8 (15%)	27 (50%)	11 (20%)	3(5%)
At 2 hours	5 (10%)	17 (31%)	16 (29%)	8 (15%)	8 (15%)	0

Among the 39 Secondary Spontaneous Pneumothorax cases, chemical pleurodesis was done within seven days in 22 (56%), whereas it was done after seven days in 17 (44%). In all cases of Primary Spontaneous Pneumothorax, pleurodesis was done by the third day.

## DISCUSSION

The present paper explores the role of iodopovidone as an agent for chemical pleurodesis. When medical thoracoscopy is not easily available in our country, there are limited options for choosing agents for chemical pleurodesis.

An ideal sclerosing agent should have a high molecular weight and chemical polarity, low regional clearance, rapid systemic clearance, a steep dose-response curve and should be well tolerated with minimal or no side-effects.^7^ At the same time, the agent should be easily available, widely accepted, cheap and effective.

Though talc is the most widely used agent worldwide, ARDS complications have been encountered with the use of talc, which occurs especially if the talc used has a smaller particle size (< 15μm).^8^ At the same time, medical-grade talc is not commercially available in Nepal. Though IV Tetracycline has been used in other countries, this agent again is not available commercially. In such a scenario, iodopovidone, if found effective, would be a beneficial agent in a resource-constrained setting like Nepal for chemical pleurodesis.

In our study, the most common diagnoses were COPD, Post TB sequelae and malignant pleural effusion. The majority of cases were understandably more than 60 years of age.

In the case of secondary spontaneous pneumothorax, complete response was observed in 90% of the cases, whereas treatment failed in 5% cases. In a retrospective analysis of thoracoscopic instillation of iodopovidone in patients with primary spontaneous pneumothorax, the overall success rate was 94%.^9^ Agarwal et al.^10^ obtained a complete response rate of 92.6% in the pneumothorax group with povidone-iodine in a study including 27 patients with pneumothorax. In both the studies, pleurodesis was done via tube thoracostomy as was done in our study. The rate of success achieved in our study is similar to the other studies. In cases of primary spontaneous pneumothorax, complete response was achieved in 100% of the cases.

In the case of malignant pleural effusion, complete response was achieved in 55% cases, whereas the treatment failed in 45%. In a multicenter study of malignant pleural effusions, in which thoracoscopy had been the most common procedure, a complete response was obtained in 96% of patients.^11^ In another study, this procedure's response was complete in 72.2% patients and partial in 19.4% of patients. Treatment failure was displayed in 8.3% of patients.^1^ malignant pleural effusion (MPE2) Similar to our study, pleurodesis was performed via the thoracostomy tube in this study. The response rate is expectedly higher when pleurodesis is performed during thoracoscopy, as pleural adhesions and fluid loculations can be easily eliminated.^10^

In the single case of hepatic hydrothorax, pleurodesis was successfully performed with iodopovidone. There have been various reports of success of pleurodesis in cases of hepatic hydrothorax.^13,14^

In a review of six studies, 265 patients underwent chemical pleurodesis with iodopovidone, and the mean success rate was 90.6%. In this meta-analysis, pleurodesis with iodopovidone was performed for recurrent pleural effusion in 157 patients and pneumothorax in 108 patients.^6^ In a review of different pleurodesis agents, the overall complete response was obtained in 60.4% of 1,168 patients. Talc had the highest complete response rate at 93%, followed by Tetracycline (67%) and Bleomycin (54%).^1^ In our present study, the efficacy of iodopovidone is comparable to that of talc in pneumothorax cases. In contrast, the lesser success rate in malignant pleural effusion cases could be attributed to pleurodesis done via tube thoracostomy in our case compared to thoracoscopy.

Iodine has strong oxidative and cytotoxic properties, which can induce a potent inflammatory response in the wall of any fluid-containing structure.^15^ This, coupled with the low pH (2.97) of the sclerosing solution, may lead to pleurodesis.^11^

In the review by Walker et al.,^1^ the most commonly reported adverse effects were pain (265 of 1140, 23%) and fever (220 of 1140, 19%). Another study reported that all the patients in their study experienced chest pain; the other side effects reported were fever in seven patients and empyema in one patient.^10^ Serious chest pain and hypotension were detected in 5.8% of the cases in another study.^11^ In the study by Godazandeh et al.,^12^ malignant pleural effusion (MPE the most common complaints of patients were pain (n=14, 35.9%) followed by dyspnea (n=8, 20.5%), burning (n=7, 17.9%) and fever (n=3,7.7%). In our study, various degree of pain was present in all cases. Besides pain, the burning sensation was present in 78%, followed by dyspnoea in 19% and fever in 4%. There were no serious side effects of the procedure. Thyroid function was not done before pleurodesis or after pleurodesis. No changes in thyroid function were detected before and one week after pleurodesis with iodopovidone in another study.^12^ malignant pleural effusion (MPE Thus, the use of iodopovidone without prior thyroid function is also a viable alternative, and the side effect profile of iodopovidone is manageable.

## CONCLUSIONS

The high complete response rate achieved with iodopovidone for chemical pleurodesis done via tube thoracostomy coupled with the fact that the agent is safe, cheap and easily accessible makes iodopovidone the agent of choice for chemical pleurodesis in Nepalese context.

## References

[1] Walker-Renard PB, Vaughan LM, Sahn SA (1994). Chemical pleurodesis for malignant pleural effusions. Ann Intern Med.

[2] Kilic D, Findikcioglu A, Hatipoglu A (2006). A different application method of talc pleurodesis for the treatment of persistent air leak. ANZ J Surg.

[3] Berger R (1994). Pleurodesis for spontaneous pneumothorax. Will the procedure of choice please stand up? Chest.

[4] Melvin WS, Krasna MJ, McLaughlin JS (1992). Thoracoscopic management of spontaneous pneumothorax. Chest.

[5] Menzies R, Charbonneau M (1991). Thoracoscopy for the diagnosis of pleural disease. Ann Intern Med.

[6] Agarwal R, Khan A, Aggarwal AN, Gupta D (2012). Efficacy &amp; safety of iodopovidone pleurodesis: a systematic review & amp; meta-analysis. Indian J Med Res.

[7] Antunes G, Neville E, Duffy J, Ali N (2003). Pleural Diseases Group, Standards of Care Committee, British Thoracic Society. BTS guidelines for the management of malignant pleural effusions. Thorax.

[8] Maskell NA, Lee YCG, Gleeson FV, Hedley EL, Pengelly G, Davies RJO (2004). Randomized Trials Describing Lung Inflammation after Pleurodesis with Talc of Varying Particle Size. Am J Respir Crit Care Med.

[9] Estrada Saló G, Farina Ríos C, Fibla Alfara JJ, Gómez Sebastián G, Unzueta MC, León González C (2003). Spontaneous pneumothorax: pleurodesis with an iodo-povidone hydroalcoholic solution. Arch Bronconeumol.

[10] Agarwal R, Aggarwal AN, Gupta D (2006). Efficacy and safety of iodopovidone pleurodesis through tube thoracostomy. Respirology.

[11] Olivares-Torres CA, Laniado-Laborín R, reo Chá vez-García C, Leó n-Gastelum C, Reyes-Escamilla A, Light RW (2017). Iodopovidone Pleurodesis for Recurrent Pleural Effusions. Chest.

[12] Godazandeh G, Qasemi NH, Saghafi M, Mortazian M, Tayebi P (2013). Pleurodesis with povidone-iodine, as an effective procedure in management of patients with malignant pleural effusion. J Thorac Dis.

[13] Lee WJ, Kim HJ, Park JH, Park D Il, Cho YK, Sohn C Il (2011). Chemical pleurodesis for the management of refractory hepatic hydrothorax in patients with decompensated liver cirrhosis. Korean J Hepatol.

[14] Boin IF, Silva AM, Leonardi LS (2001). Chemical pleurodesis for hepatic hydrothorax. Arq Gastroenterol.

[15] Brissaud O, Desfrere L, Mohsen R, Fayon M, Demarquez JL (2003). Congenital idiopathic chylothorax in neonates: chemical pleurodesis with povidone-iodine (Betadine). Arch Dis Child Fetal Neonatal Ed.

